# HIV knowledge, risky behaviours and public health care services attendance among adolescents from the Grassroot soccer Zimbabwe programme

**DOI:** 10.1186/s12913-020-05305-3

**Published:** 2020-05-13

**Authors:** Mayibongwe Louis Mzingwane, Greanious Alfred Mavondo, Fennie Mantula, Claudinos Mapfumo, Calleta Gwatiringa, Bhekimpilo Moyo, Primrose Dube, Cynthia Nombulelo Chaibva

**Affiliations:** 1 National University of Science and Technology, Faculty of Medicine, Bulawayo, Zimbabwe; 2 Department of Pathology, National University of Science and Technology, P. O Box AC939, Ascot, Bulawayo, Zimbabwe; 3 Grassroot Soccer Zimbabwe, Bulawayo, Zimbabwe

**Keywords:** HIV, Adolescents, Knowledge, Risky behaviours, Health care

## Abstract

**Background:**

Interventions aimed at improving accessing of health care services, including HIV testing, remain a priority in global HIV eradication efforts. Grassroot Soccer Zimbabwe (GRSZ) is an adolescent health organisation that uses the popularity of soccer to promote healthy behaviours. We assessed HIV knowledge levels, risky behaviours and attitudes in school going adolescents and young adults who attended GRSZ programmes and determined if HIV knowledge levels were associated with increased levels of accessing of health care services by youths.

**Methods:**

This was a cross-sectional study carried out on 450 participants aged 13–30 years who attended at least one of the three programmes offered by GRSZ. Self-administered and self-reporting questionnaires were used to collect information on participants’ demographics, knowledge on HIV and reproductive health, sources of information, access to HIV and reproductive health services and attitudes and risky behaviours.

**Results:**

A total of 392 (87.1%) responses were received. High HIV and reproductive health knowledge levels were recorded (77.7%) on our scale with females recording higher levels (81.1%) than males (71.1%). The majority of participants (72%) indicated willingness to abstain from risky behaviours such as use of drugs and attending youth sex parties. However about 33.3% of the participants who had sexual intercourse reported having condomless sex. There was marginal association between high HIV and reproductive health knowledge levels and accessing health care services in the past 24 months (*p* = 0.045).

**Conclusions:**

HIV and reproductive health knowledge levels were relatively high among adolescents and were associated with accessing health care services in the past 24 months. There however are some gaps associated with engaging in risky sexual behaviours such as condomless sex which could be addressed by using these findings to assist organizations working with adolescents, educators and policy makers in developing programmes that address adolescent sexual behaviours.

## Plain English summary

Efforts aimed at ending HIV infections require that people regularly visit health care institutions for services such as HIV testing. Grassroot Soccer Zimbabwe (GRSZ) is an adolescent health organisation that uses the popularity of soccer to promote healthy behaviours. We assessed HIV knowledge levels, risky behaviours and attitudes in school going adolescents and young adults who attended GRSZ programmes and determined if HIV knowledge levels were associated with increased levels of accessing of health care services by youths. In this study we collected information from 450 individuals aged 13–30 years who had attended at least one of the three programmes offered by GRSZ. We collected information on demographics of the participants, their knowledge on HIV and reproductive health, sources of information, access to HIV and reproductive health services and attitudes and risky behaviours. A total of 392 (87.1%) responses were received. High HIV and reproductive health knowledge levels were recorded (77.7%) on our scale with females recording higher levels (81.1%) than males (71.1%). The majority of participants (72%) indicated willingness to abstain from risky behaviours such as use of drugs and attending youth sex parties. However about 33.3% of the participants who had sexual intercourse reported having condomless sex. Our results indicate that individuals who had sought health-care services in the past 24 months had higher HIV and reproductive health knowledge levels compared to those who had not. Participants indicated willingness to abstain from risky behaviours although some risky sexual practises such as condomless sex were reported indicating a need to address such gaps. The findings of the study may assist organizations working with adolescents, educators and policy makers in developing programmes that address adolescent needs aimed at improving their health and eradicating HIV.

## Background

Adolescent girls and young women aged 15–24 years, who account for just 11% of the adult population, accounted for 20% of new HIV infections among adults globally in 2015 compared to 14% in young men of the same age group [1]. In Zimbabwe about 13.5% of young people aged 13–24 years are living with HIV [2] and only 45% of young men and 62% of young women have ever tested for HIV [3]. Additionally, only 46% of young women and 47% of young men have comprehensive knowledge about HIV [3], as a result they are unlikely to seek HIV testing and prevention services.

Global co-operation and strategies are needed to end the HIV epidemic. In 2014, the United Nations programme on HIV/AIDS introduced a goal to end the AIDS epidemic by 2030 and a programme referred to as the 90–90-90 concept is part of the plan towards achieving this goal [4]. It is targeted that by 2020, 90% of all people living with HIV should know their HIV status, 90% of all people diagnosed with HIV should be on combined antiretroviral therapy (cART) and that 90% of all people receiving cART will should virally suppressed. Currently, approximately 40% of people living with HIV globally do not know their status. There is still a large number of people living with HIV who are not on cART and only 45% of those on cART are virally suppressed. Clearly, achieving the first 90 is the first step towards the success of this concept. Interventions aimed at improving accessing of health care services including HIV testing should therefore be promoted particularly for the 15–24 years high risk age group.

Grassroot Soccer Zimbabwe (GRSZ) is an adolescent health organisation that uses the popularity of soccer to promote healthy behaviours and educate youth in areas such as HIV, healthy relationships, multiple partners, intergenerational sex, and gender based violence. Entertainment has been used as an educational tool for HIV intervention in other programmes [5]. In GRSZ programmes soccer is used as a tool to mobilize boys and girls to attend their programmes where education and interventions aimed at improving knowledge of risky behaviours and reducing the spread of HIV and AIDS are given. A similar intervention has previously been used on primary school children [6]. In the GRSZ programme, three interventions known as Generation SKILLZ, SKILLZ street and SKILLZ holiday are offered and target adolescents aged 13–19 years. Generation SKILLZ focuses on promoting healthy relationships among youths. SKILLZ Street is for girls only and aims to empower girls to stand against gender based violence. SKILLZ Holiday is delivered over school holidays and addresses risky behaviours that promote the spread of HIV, while promoting healthy behaviours. The GRSZ programmes have not been evaluated since their inception in 2011.

In this study we assessed HIV knowledge levels, behaviours and attitudes in school going adolescents and young adults who had attended GRSZ programmes and gauged the impact of such programmes as an HIV intervention method and vehicle for improving accessing of health care services among adolescents.

## Methods

### Setting

The evaluation was conducted in Bulawayo where the schools that implemented the three SKILLZ programmes are located. Eleven (11) secondary schools that have participated in the GRSZ programmes were purposively selected for the evaluation exercise (Table 1). The selection of participating schools was based on the location of the schools in relation to prevalence of risky behaviours requiring intervention. The evaluation study was run from July 2017 to February 2018.

**Table 1:** Schools selected for the evaluation exercise

School name	Location	Number of graduates	Sample size^a^
Cowdray Park High school	Cowdray Park	1346	38
Luveve High School	Luveve	1537	44
Magwegwe High School	Magwegwe	1982	56
Masotsha High School	Magwegwe	1660	47
Montrose High School	Montrose	586	17
Mzilikazi High School	Mzilikazi	2240	64
Nkulumane High School	Nkulumane	5106	27*
Sizane High School	Pelandaba	2958	18*
Sobukazi High School	Mzilikazi	2221	63
St Bernards High School	Pumula	992	28
Pumula High School	Pumula	1695	48
**Total**		22,323	450

^a^The figures were calculated using number of graduates from St Columbus High School (930) and Founders High school (608) who were initially targeted but later replaced by Nkulumane High school and Sizane High school respectively

### Study population and sampling

The population comprised of school going adolescents that participated in the SKILLZ programmes. The sample size per school was based on the number of SKILLZ graduates from that school (Table 1). A total sample of size of 383 was calculated using EPI-Info at 95% confidence level and 5% confidence interval and increased to 450 to accommodate non-responses.

### Data collection

Self-administered and self-reporting paper based questionnaires (additional file) were distributed on-site using research assistants and collected soon after completion to a sample of 450 adolescents who attended at least one of the three programmes. The adolescents were non-random and consecutively recruited from the 11 purposively selected schools that participated in the three SKILLZ programmes (Table 1).

### Questionnaire creation and validation

The questionnaire was adapted from various questionnaires from similar studies [7–9]. Questions relevant to the study were chosen and adapted to the local situation. The questions were reviewed to establish face validity after which the questionnaire was piloted in forty adolescents from two schools that had participated in the three SKILLZ programmes but were not part of the study sites as indicated in Table 1.

### Measures

The questionnaire had four sections. Section A assessed demographic characteristics (Sex and age), education attainment goals and family type (Single parent, both parents, guardian, siblings only) and size (Number of siblings). Section B had 10 questions covering reproductive health and HIV transmission, prevention and treatment to measure HIV Knowledge levels. Participants were asked to indicate if each question was ‘True’, ‘False’ or ‘I don’t know’. Knowledge level score was categorized as ‘high’ for a score greater than 63% and ‘low’ for a score less than 63%. Section C asked questions on sources of information on HIV and puberty and accessing of health care services. Participants were asked the questions ‘Do you find it difficult or easy to talk to your parents about things that you consider important to you?’ and, ‘Have you ever discussed sex-related matters with your parents or guardian?’ They were then asked to indicate from a provided list in each case what had been important sources of information on puberty for them, which health care service providers they had accessed in the past 24 months and the services accessed. Section D measured attitudes and risky behaviours. Participants were asked to indicate the social activities they like attending or taking part in from a list that included the following: Youth sex parties, nightclubs, sporting activities and other activities that they had to specify. The next set of questions required participants to answer, ‘YES’ or ‘NO’ to the questions, ‘Do you ever drink alcohol?’, ‘Do you ever smoke cigarettes or take other drugs such as marijuana (mbanje) and others?’, and ‘Have you ever had sexual intercourse?’. Participants who indicated ‘YES’ to ever had sexual intercourse were to respond to further questions covering age at first sexual intercourse, age of sexual partners, last time they had had sexual intercourse and protection method used against infections and pregnancy. Self-efficacy to abstain from sexual intercourse was assessed by asking the question, ‘What are your reasons for not having intercourse?’ and to choose a statement that best describes their future plans about sexual intercourse. Lastly participants were to indicate if they, ‘strongly agree’, ‘agree’, ‘disagree’ or ‘strongly disagree’ with the following statements: ‘It is OK for people my age to have sex with different partners’, ‘Condoms should be used if a person my age is having sex’, ‘Engaging in sexual intercourse at my age makes me popular’ and ‘There is nothing wrong with unmarried boys and girls having sexual intercourse if they love each other’.

### Data management and analysis

The outcome variables were: HIV and reproductive health knowledge levels recorded as high or low, Sources of information on HIV and puberty as a categorical variable, Health care services accessed in the past 24 months measured as categorical variable and Attitudes and risky behaviours measured as a binary variable. These were measured against Sex, age, family type and the level of education participants expected to reach as independent variables. Univariate analysis was conducted to come up with frequencies and proportions for categorical variables. Bivariate analysis was done to test associations between demographic characteristics and knowledge levels and also between access to health care services and knowledge levels.

### Ethics and consent

Ethical approval was obtained from the Medical Research Council of Zimbabwe (Ref. A/2226). Additional approvals were obtained from the Ministry of Education and the participating schools. Informed consent was obtained from all the adolescents and their parents. The questionnaire was completed privately and did not contain any personally identifiable information.

## Results

### Demographic characteristics

A total of 392 responses were received from 450 questionnaires distributed, a response rate of 87.1%. The majority of respondents were female (76.3%) and most respondents belonged to the 15–19 years age group (78.3%). Table 2 shows the distribution of the demographic variables (gender, age, education attainment goals and family type).

**Table 2:** Demographic characteristics of respondents [*n* = 392]

Variable	n (%)
**Sex**	
Male	92 (23.5)
Female	299 (76.3)
Not reported	1 (0.3)
**Level of education expected to reach**	
High school	20 (5.1)
College	12 (3.1)
University	359 (91.6)
Not reported	1 (0.3)
**Family type**	
Single parent headed	101 (25.8)
Staying with both parents	167 (42.6)
Staying with a guardian	107 (27.3)
Siblings staying alone	17 (4.3)
Average family size(SD)^a^	4 (1.6)
**Age group**	
13–14	65(16.6)
15–19	307(78.3)
20–24	10(2.6)
25+	10(2.6)

^a^*SD* Standard deviation

### Sources of information and reproductive health services

The school teacher was the most frequently picked source of information on puberty (62.2%) and GRSZ was the third most frequently picked source (55.9%). Health facility was also included in the list and about a quarter of the respondents (24.5%) picked it as an important source of information on puberty. Parents were cited as important sources of information by 66.2% of the respondents with the majority of participants in Generation SKILLZ (57.9%) and SKILLZ street (61.6%) reporting that it was easy for them to talk to parents about issues that are important to them.

**Fig. 1:**
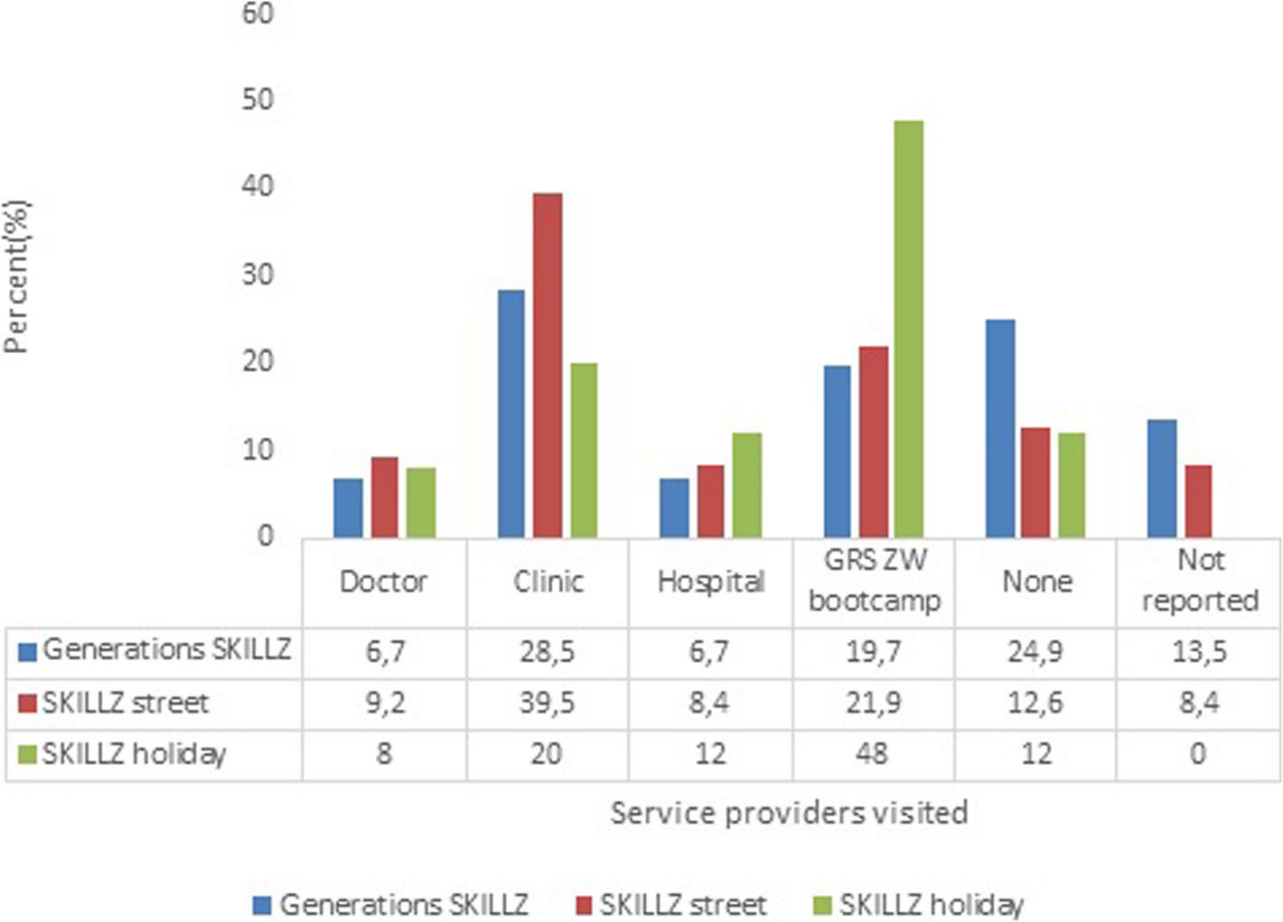
Service providers visited in the past 24 months for information and reproductive health services

The clinic was the health service provider that was most frequently visited by respondents from Generation SKILLZ and SKILLZ Street programmes (28.5 and 39.5% respectively) while GRSZ boot camp provided health care services for 48% of participants from SKILLZ holiday (Fig. 1). Voluntary medical male circumcision (VMMC) and voluntary HIV counselling and testing (VCT) were the services mostly accessed by respondents who attended Generation SKILLZ (26.8%) and SKILLZ holiday (46.1%) programmes while SKILLZ Street participants mostly accessed health care service providers for information on STIs (Table 3).

**Table 3:** Services accessed from health care service providers in the past 24 months

Service	Generations SKILLZ	SKILLZ Street	SKILLZ Holiday
	n (%)	n (%)	n (%)
Contraception	11 (4.45)	14 (7.2)	1 (3.9)
Voluntary HIV counseling and testing (VCT)	31 (12.6)	15 (7.7)	5 (19.2)
Sexually transmitted infection	29 (11.7)	29 (14.9)	2 (7.7)
Pregnancy test	7 (2.8)	5 (2.6)	4 (15.4)
Pregnancy termination	5 (2.0)	4 (2.1)	1 (3.9)
Medical male circumcision	35 (14.2)	0	7 (26.9)
Other	44 (17.8)	29 (14.9)	2 (7.7)
Not reported	85 (34.4)	99 (50.8)	4 (15.38)

### HIV and reproductive health knowledge levels

HIV and reproductive health knowledge levels were ‘high’ for 77.7% of the participants. More females (81.1%) had higher knowledge of HIV and reproductive behaviour than males (71.1%). Among participants categorized as having ‘low’ HIV and reproductive health knowledge levels, siblings staying alone constituted the highest proportion (41.7%). We wanted to know if there was an association between participants’ knowledge levels and demographic variables measured. The test of association between ‘knowledge level of HIV and reproductive health’ and the variables was as follows: Participants’ sex (*χ*^2^ =3.19, *p* = 0.07), family type as categorized in Table 2 (*χ*^2^ =3.0, *p* = 0.40) and age of the participants (*χ*^2^ =2.6, *p* = 0.45). HIV and reproductive health knowledge levels were however associated with accessing health care services in the past 24 months (*p* = 0.045).

### Attitudes and behaviours related to risk of HIV and STI acquisition

**Fig. 2:**
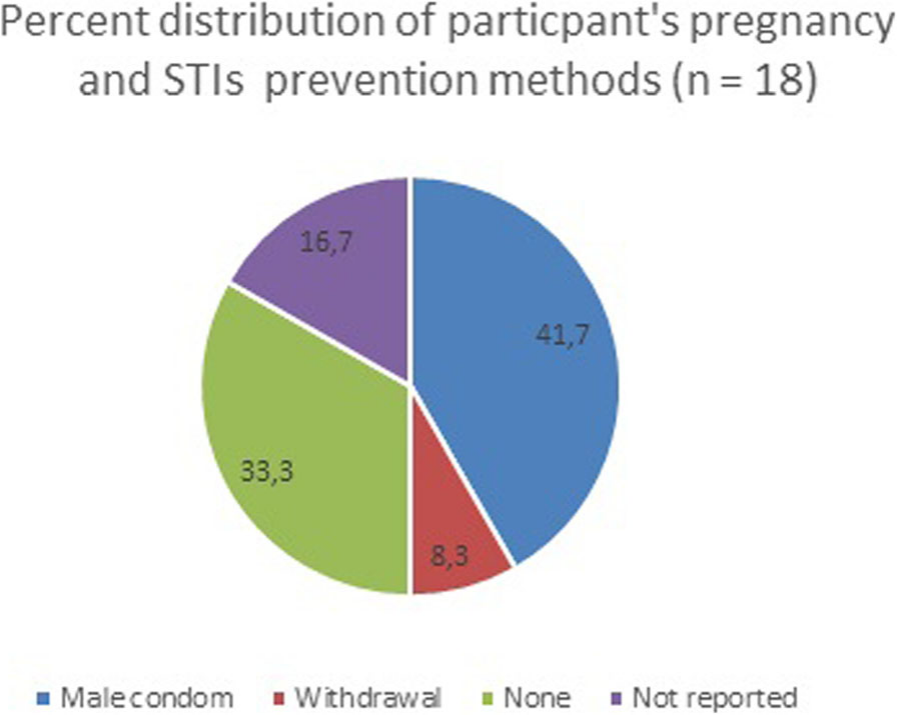
Pregnancy and STIs prevention methods used by participants who have engaged in sexual intercourse

The majority of participants (72%) demonstrated willingness to abstain from risky activities such as youth sexual parties (known as Vuzu parties) and also reported abstaining from drugs and alcohol. Figure 2 shows percentage distribution of participants who had engaged in sexual intercourse and used prevention methods for STIs and pregnancy. A total of 18 (5%) participants reported that they had ever had sexual intercourse and 41.7% of them revealed that they had used male condoms for both STI and pregnancy prevention. About 33.3% of the participants who had sexual intercourse reported condomless sex. There was significant association between engaging in sexual intercourse and condom use (*χ*^2^ (df = 1) = 13.78, *p*-value = 0.001) with boys more likely to report condom use than females. Only one participant from the 15–19 age group reported that she had sexual intercourse with a man 10+ years older than her and there were 15 participants, 4 males and 11 females, who had their first sexual encounter before the age of 15 years.

Most participants also showed a positive attitude against risky sexual behaviours (82, 76 and 75% for SKILLZ street, Generation SKILLZ and SKILLZ holiday respectively). They did not support the opinion of having multiple sexual partners and supported condom use if sexually active. However, males and females differed on whether it was appropriate or not for boys and girls to have sexual intercourse if they love each other with the majority of boys (55%) agreeing or strongly agreeing with the statement.

## Discussion

We report on HIV and reproductive health knowledge, attitudes and risky behaviours among adolescents and young adults in Bulawayo Zimbabwe. A self-administered questionnaire was used to gather information on participants’ knowledge levels on HIV and reproductive health related issues, their sources of information on puberty and access to HIV and reproductive health services. The questionnaire also covered attitudes and risky behaviours that may lead to acquisition of HIV and STIs including unwanted pregnancies and knowledge on methods of prevention. High HIV and reproductive health knowledge levels were recorded on our scale. It must however be noted that there are various other organizations offering programmes on HIV and reproductive health and targeting the same participants as GRSZ. A number of studies on HIV knowledge, reproductive health, risky behaviours and suggested interventions among adolescents in various parts of Zimbabwe have been reported over the years but challenges still persist [10–13]. Studies in other countries that had high HIV knowledge levels in youth attributed these to HIV educational programmes that have been rolled out over time [9, 14–16]. In Bulawayo Zimbabwe, high HIV knowledge levels could be attributed to other programmes being offered such as the School Guidance and Counselling classes, DREAMS, YES games and Trinity together with the GRSZ programme. The higher HIV knowledge levels in females compared to males have been observed in other studies in South Africa and Ghana [16, 17] while in Ethiopia males scored higher knowledge levels than females [18, 19].

Although a large number of respondents indicated that it was easy for them to talk to their parents/guardians about things that are important to them, e.g. up to 61.6% for SKILLZ Street, our data indicates that there is need for the communication to include sexual and reproduction health issues. We noted that among those who find it easy to talk to their parents, sex related topics were not discussed and the school teacher was the major source of information on puberty. One study in Cameroon also reported that 66.4% of youth aged 13–24 obtained their information on HIV from school programmes [20]. A study looking at parents’ knowledge on adolescents’ sexual reproductive health in Bulawayo suggested that parents are constrained by cultural and traditional norms, limited information and lack of skills in discussing sexual issues with their children [21]. Programmes such as those provided by GRSZ can fill up the gaps left by parents by providing platforms where adolescents can get information on puberty, ask questions and seek guidance from the life skills coaches. Another intervention might be the involvement and training of parents in communication about sexual issues and how they can provide a conducive environment needed for adolescents to freely express themselves.

There was an association between HIV and reproductive health knowledge levels and accessing health care services. The majority of participants visited health care services for HIV testing and medical male circumcision. This may indicate that intensifying HIV and reproductive health education can lead to more adolescents visiting health care institutions to access HIV testing services. Many people are reluctant to access HIV testing services with the UNAIDS reporting that approximately 40% of people living with HIV do not know their status [4]. Intensifying HIV education in youths may contribute to the current goal of eradicating HIV by 2030 and in achieving the 90–90-90 targets, particularly the first 90, as the number of people accessing HIV testing services will increase. A literature review on evidence of best practise in health services targeting adolescents revealed lack of adequate health services that address specific adolescents’ needs resulting in knowledge gaps and barriers to health services such as HIV testing [22].

The majority of participants demonstrated willingness to abstain from risky behaviours and had knowledge of the consequences of such risky behaviours although some risky sexual practises such as condomless sex were reported indicating a need to address such gaps. Intergenerational sexual relationships among our participants were low. This practice can contribute to the spread of HIV and other STIs [23–25] as younger girls may have no negotiating power in the relationship. Age at first sex is another important indicator of exposure to risk of pregnancy and STIs. Young people who initiate sex at an early age are typically at higher risk of becoming pregnant or contracting HIV and STIs than young people who initiate sex later [26, 27]. We recorded one participant reporting sexual intercourse with a man 10+ years older than her and 15 participants reporting first sexual encounter before the age of 15 years. These figures (5.5%) are lower than the 17% intergenerational sexual relationships prevalence reported in the ZDHS 2014–15 report [3]. The figures on adolescents initiating sexual intercourse before the age of 15 (3.8%) are however comparable to the ZDHS 2014–2015 figures which reported that 4.7% of young women and men in the age category 15–19 years initiate sexual intercourse before the age of 15 [3]. Participants were asked at what age they had their first sexual intercourse. Such questions are sensitive and it is possible that some respondents may have been reluctant to provide information on their sexual behaviour as only 18 out of 392 participants reported sexual intercourse. Generally, the GRSZ programme has had a great impact on participants the majority of whom plan to use the knowledge gained for empowering themselves and their communities on HIV and reproductive health issues.

There were however some limitations to the study. Firstly, because of the SKILLZ street programme which only recruits girls, the majority of participants were girls which affected analysis and sex-disaggregation of findings. Although the self-administered questionnaire allowed participants to respond anonymously, there may have been reluctance to respond to questions that directly required answers on sexual behaviour resulting in high percentage on non-responders to this issue. As the sampling was non-random, the findings may not be representative of the population being studied.

## Conclusions

HIV and reproductive health knowledge was associated with accessing health care services in the past 24 months. Intensification of HIV and reproductive health education to adolescents using youth friendly strategies such as soccer may therefore be used as a way of increasing accessing of health care services including HIV testing and voluntary medical male circumcision. There however were some gaps associated with engaging in risky sexual behaviours such as condomless sex which could be addressed by using these findings to assist organizations working with adolescents, educators and policy makers in developing programmes that address adolescent sexual behaviours. Some suggested strategies include integration of HIV and adolescents health care delivery and the use of outreach as an option for HIV testing among adolescents.

## Supplementary information

**Additional file 1.** Participant questionnaire: Grassrootsoccer Zimbabwe (GRS ZW) SKILLZ programmes evaluation. Description of data: Questionnaire

## Data Availability

The datasets used and/or analysed during the current study are available from the corresponding author on reasonable request.

## Abbreviations

cART: Combined antiretroviral therapy
DREAMS: Determined, Resilient, Empowered, AIDS-free, Mentored and Safe
GRSZ: Grassroot Soccer Zimbabwe
SPSS: Statistical Package for Social Sciences
STATA: Statistics and data
STIs: Sexually transmitted infections
UNAIDS: United Nations programme on HIV/AIDS
VCT: Voluntary HIV counselling and testing
VMMC: Voluntary medical male circumcision
ZDHS: Zimbabwe demographic and Health survey
