# Supplementary material for: HIV knowledge, risky behaviours and public health care services attendance among adolescents from the Grassroot soccer Zimbabwe programme
Source: BMC Health Serv Res. 2020 May 13;20:420. doi: 10.1186/s12913-020-05305-3 (PMC7222321; doi:10.1186/s12913-020-05305-3)
Supplement: Supplementary file 1 — Additional file 1. Participant questionnaire: Grassrootsoccer Zimbabwe (GRS ZW) SKILLZ programmes evaluation. Description of data: Questionnaire [file 12913_2020_5305_MOESM1_ESM.docx]

**Participant questionnaire: Grassrootsoccer Zimbabwe (GRS ZW) SKILLZ programmes evaluation**

Instructions: Please respond to all questions by either ticking on the boxes or writing on the spaces provided

**Section A: Demographics**

| **1. Gender:** Male Female | **2. Date of birth:**  Day Month Year |
| --- | --- |
| **3. What form are you currently in at this school?**    1 2 3 4 5 6 I am a former student | |
| **4. What is the highest level of education that you expect to reach:**  Secondary school High school College University | |
| **5. Who do you stay with at home?**  one parent both parents guardian siblings only alone | |
| **6. How many children are there in your family? _______________________** | |
| **7. In which of the Grassrootsoccer Zimbabwe (GRS ZW) programme or programmes**  **did you participate?**  Generation SKILLZ  SKILLZ street  SKILLZ holiday | |

**Section B: Knowledge of HIV and reproductive health**

For each statement below please circle “True” (T), “False” (F) or “I don’t know’’ (DK)

|  | **True** | **False** | | **I don’t know** | |
| --- | --- | --- | --- | --- | --- |
| 1. **Coughing and sneezing can spread HIV.** | T | | F | | DK |
| 1. **Washing one’s genitals or private parts after sex can protect you from getting HIV.** | T | | F | | DK |
| 1. **An HIV-infected man can be cured of HIV if he has sex with a young girl who is a virgin.** | T | | F | | DK |
| 1. **People who have been infected with HIV always look unhealthy.** | T | | F | | DK |
| 1. **There is a female condom that can help reduce a woman’s chance of getting HIV.** | T | | F | | DK |
| 1. **Having sex with more than one partner can increase a person’s chance of being infected with HIV.** | T | | F | | DK |
| 1. **Taking a test for HIV one week after having sex will tell a person if he or she has HIV.** | T | | F | | DK |
| 1. **A woman can get pregnant on the very first time that she has sexual intercourse.** | T | | F | | DK |
| 1. **Girls cannot get HIV from young boys who have had sex only a few times with other partners.** | T | | F | | DK |
| 1. **HIV can be transmitted by sharing needles or syringes.** | T | | F | | DK |

**Section C: Sources of information and reproductive health services**

| **18. Do you find it difficult or easy to talk to your parents about things that you consider important to you?** Difficult Easy Not sure |
| --- |
| **19. Have you ever discussed sex-related matters with your parents or guardian?**  Yes No |
| **20. Which of the following have been important sources of information for you on puberty**  **(the ways in which boys’ and girls’ bodies change during the teenage years)?**  School teacher Mother Father  Other family members Sister Brother  Health facility Friends GRS ZW Media (TV/Radio/Books/Facebook) Other (Specify……………………) |
| **21. Which of the following have you visited in the past 24 months to access services or information on contraception, pregnancy, abortion or sexually transmitted diseases?**  Doctor Clinic Hospital GRS ZW bootcamp  Other (Specify…………………………….)  **22. What were the services accessed?**  Contraception VCT Sexually transmitted infection  Pregnancy test Pregnancy termination Medical male circumcision  Other (Specify ……………………………………………..) |

**Section D: Attitudes and behaviours**

| **23. Which of the following social activities do you like attending or taking part in?**  Youth sex Parties (Vuzu) Nightclubs Soccer  Other sporting activities Other activities (specify)___________________ | | | | |
| --- | --- | --- | --- | --- |
| **24. Do you ever drink alcohol?** Yes No | | | | |
| **25. Do you ever smoke cigarettes or other drugs such as marijuana (mbanje) or other**  **drugs**? Yes No | | | | |
| **26. Have you ever had sexual intercourse?** Yes No (NB: Sexual  intercourse refers to insertion of a man's erect penis into a woman's vagina.)  **If you answered “NO” to question 27, please proceed to question 36**  **27. If “YES”. How old were you when you had your first sexual encounter? __________**  **28. How old was the person you first had sex with? _______________________________**  **29. When was the last time you had sexual intercourse? ___________________________**  **30. How old was the person you last had sex with? _______________________________**  **31. What protection did you use to prevent pregnancy?**  Male condom Female condom Birth control pill Implant  Injection withdrawal Other (Specify …………………)  **32. What protection did you use to prevent sexually transmitted infections?**  Male condom Female condom Other (Specify ……………………) | | | | |
| **33.** **How many of your friends have had sexual intercourse?**  Many A few None Not sure | | | | |
| **34. If you answered “NO” to question 31. What are your reasons for not having**  **intercourse? Please tick all that apply to you.**  I am not ready to have sex  I have not had the opportunity to have sex  I think that sex before marriage is wrong  I am afraid of getting pregnant/ impregnating  I am afraid of getting HIV or other sexually transmitted infections  I Di | | | | |
| **35. Which of these statement best describe your future plans about sexual intercourse**  **regardless of whether you have had sexual intercourse or not?**  I plan to wait until marriage  I plan to stop engaging in sexual intercourse  I plan to continue with sexual intercourse  I plan to wait until I am engaged to be married  I plan to wait until I find someone I love  I plan to have sexual intercourse when I get an opportunity  I plan to use the knowledge gained from GRS ZW | | | | |
| **Please indicate if you “Strongly agree” (1), “Agree” (2), “Disagree” (3) or “Strongly**  **Disagree” (4) with the following statements** | | | | |
|  | **1** | **2** | **3** | **4** |
| **36.** It is OK for people my age to have sex with different partners |  |  |  |  |
| **37.** Condoms should be used if a person my age is having sex |  |  |  |  |
| **38.** Engaging in sexual intercourse at my age makes me popular. |  |  |  |  |
| **39.** There is nothing wrong with unmarried boys and girls having  sexual intercourse if they love each other |  |  |  |  |
